# Comparison of Amyloid-PET Analysis Software Using ^18^F-Florbetaben PET in Patients with Cognitive Impairment

**DOI:** 10.3390/diagnostics15162028

**Published:** 2025-08-13

**Authors:** Miju Cheon, Hyunkyung Yi, Sang-Won Ha, Min Ju Kang, Da-Eun Jeong, Yasser G. Abdelhafez, Lorenzo Nardo

**Affiliations:** 1 Department of Nuclear Medicine, Veterans Health Service Medical Center, Seoul 05368, Republic of Korea; 2 Department of Neurology, Veterans Health Service Medical Center, Seoul 05368, Republic of Korea; 3 Department of Radiology, University of California Davis, Sacramento, CA 95817, USA

**Keywords:** Alzheimer’s dementia, quantification, amyloid PET, ^18^F-florbetaben

## Abstract

**Background/Objectives**: Quantitative analysis of amyloid PET imaging plays a crucial role in diagnosing Alzheimer’s disease (AD), particularly in cases where visual interpretation is equivocal. Multiple commercial software tools are available for this purpose, yet differences in their quantification and diagnostic performance remain understudied, especially for Neurophet SCALE PET. **Methods**: We retrospectively analyzed ^18^F-florbetaben PET/CT scans from 129 patients with cognitive impairment, comprising 39 patients with AD and 90 with non-AD diagnoses, using three software tools: MIMneuro, CortexID Suite, and Neurophet SCALE PET. Standardized uptake value ratios (SUVRs) were obtained for six brain regions known for amyloid accumulation. Diagnostic accuracy was evaluated using ROC curve analysis, while inter-software correlations and reliability were assessed via Pearson correlation coefficients and intraclass correlation coefficients (ICC). **Results**: All three software programs significantly distinguished AD from non-AD patients in most brain regions. MIMneuro and Neurophet SCALE PET demonstrated the highest diagnostic performance, with MIMneuro achieving an AUC of 1.000 in the anterior cingulate gyrus. While MIMneuro and Neurophet SCALE PET showed moderate-to-strong SUVR correlations (r = 0.715–0.865), CortexID Suite showed limited correlation with the other tools. Inter-software reliability was moderate only in selected regions (ICC ≈ 0.5), indicating potential variability in SUVR measurements across platforms. **Conclusions**: MIMneuro, CortexID Suite, and Neurophet SCALE PET are effective for the semi-quantitative analysis of amyloid PET and can aid in the diagnosis of AD. However, clinicians should be cautious when interpreting SUVRs across different software tools due to limited inter-software consistency. Standardization efforts or consistent use of a single platform are recommended to avoid diagnostic discrepancies.

## 1. Introduction

Alzheimer’s disease (AD) is the most common cause of dementia and is characterized by progressive cognitive and functional decline associated with underlying neuropathological changes. The hallmarks of AD pathology include extracellular β-amyloid plaques and intracellular neurofibrillary tangles composed of hyperphosphorylated tau protein. These pathological changes lead to synaptic dysfunction, neuronal loss, and brain atrophy [1].

Today, we can diagnose AD during life with greater certainty by using a combination of tests that may include those for biomarkers. Biomarkers can detect the presence of plaques and tangles in the brain. Advances in biomarker research have enabled the in vivo detection of these pathological hallmarks, allowing for more accurate diagnosis and staging of AD. Biomarker tests include specific types of positron emission tomography (PET) scans of the brain. Newer PET imaging tests have been developed using ligands that bind to amyloid plaques and neurofibrillary tangles, which can help detect specific brain changes associated with AD. We can use PET to help diagnose AD and to determine if the disease is in the early or later stages. Among them, amyloid PET, which can measure β-amyloid deposition, is more widely used in actual clinical settings. Amyloid PET imaging has emerged as a key biomarker tool, capable of visualizing cerebral β-amyloid deposition with high sensitivity and specificity [2,3]. Several radiopharmaceuticals for PET can be used to measure β-amyloid. ^18^F-florbetaben is widely used and approved for detecting amyloid plaques in the evaluation of cognitive disorders [4].

Interpretation of amyloid PET imaging can be performed through visual reads or semi-quantitative analysis. One meta-analysis found that a pooled analysis of 48 studies involving over 5900 participants indicated an overall diagnostic performance of Aβ PET of ~90% sensitivity and 80% specificity, with visual assessment slightly outperforming quantitative methods [5]. While visual assessment is the standard method in many clinical environments due to its simplicity and established diagnostic criteria [6], there are several challenges of visual interpretation of amyloid PET scans including inter-rater variability, [7,8], difficulty to accurately interpret, especially in older individuals with cortical atrophy [9], and difficulty in making binary decision in early or borderline cases [10]. On the contrary, quantitative or semi-quantitative approaches offer better sensitivity, and some studies showed improved inter-rater agreement when combined with visual interpretation [11].

To enhance consistency and improve diagnostic accuracy, several automated image analysis software tools have been developed. These tools generate standardized uptake value ratios (SUVRs), compare regional brain uptake to reference areas, and often produce z-score maps to aid in clinical interpretation [12]. Currently, several commercial software solutions for amyloid PET quantification exist, including PMOD, CapAIBL, NiftyPET, ELBA, AmyPype, MIMneuro, BTXamyloid, CortexID Suite, and Neurophet SCALE PET, among others. These tools employ proprietary algorithms for brain segmentation, region-of-interest (ROI) extraction, and SUVR calculation. However, automated semi-quantitative analysis programs also have their drawbacks. They include differences in image processing pipelines, anatomical templates, and reference region selection (e.g., cerebellum or pons), which can lead to variability in quantitative outputs and potentially impact clinical interpretation [13,14].

Previous studies have investigated the reliability and concordance of some of these software platforms, yielding mixed findings that often vary depending on the tracer used, analysis method, and clinical context [15,16,17,18,19,20,21]. Some investigations have demonstrated strong agreement among tools in detecting amyloid positivity, while others have highlighted discrepancies in regional SUVR values and subsequent diagnostic classifications. For instance, differences in quantifying uptake in regions such as the posterior cingulate or inferior frontal cortex have been associated with discordant results. These variations may influence diagnostic decisions and patient eligibility for disease-modifying therapies. Additionally, various brain amyloid deposition measurement software programs utilize different normal databases, resulting in differences in the result values between them. This can ultimately lead to confusion in interpretation in patients with early stages of brain amyloid deposition, as well as low reproducibility and reliability. As amyloid PET imaging becomes increasingly integrated into routine clinical practice and plays a critical role in identifying candidates for anti-amyloid therapies, understanding the variability and diagnostic impact of different analysis software is essential. In particular, while several studies have compared widely used amyloid PET quantification tools, there is limited literature directly comparing MIMneuro, CortexID Suite, and Neurophet SCALE PET in the context of ^18^F-florbetaben PET within a real-world memory clinic cohort, particularly with a focus on head-to-head performance in clinical diagnosis. Also, our study includes Neurophet SCALE PET, a relatively newer tool with expanding international use but limited peer-reviewed comparative evaluation. To date, only one study has included Neurophet SCALE PET in a comparative context, focusing on Centiloid scaling but not evaluating regional SUVR values [22]. Given the lack of comprehensive comparison involving Neurophet SCALE PET, particularly in real-world clinical populations, our study aims to address this point. We conducted a head-to-head evaluation of three widely available amyloid PET quantification software tools using ^18^F-florbetaben PET data. These tools were selected based on their regulatory approvals, commercial availability, established or emerging use in clinical practice, and accessibility at our institution.

This study aims to evaluate the inter-software differences, correlation, reliability, and diagnostic performance of three commercially available software tools—MIMneuro, CortexID Suite, and Neurophet SCALE PET—for analyzing ^18^F-florbetaben PET/CT scans from patients with cognitive impairment.

## 2. Materials and Methods

### 2.1. Subjects

This retrospective study was approved by the Institutional Review Board (IRB) of [BOHUN 2023-12-020] on 19 December 2023, and the requirement for written informed consent was waived due to the retrospective design. We reviewed the medical records of 156 consecutive patients who underwent ^18^F-florbetaben PET/CT imaging between October 2020 and June 2021 at our institution. The inclusion criteria were as follows: (1) Presence of subjective or objective cognitive impairment. (2) Availability of clinical follow-up data for at least six months following PET/CT imaging. Specifically, patients were considered to have objective cognitive impairment if they met at least one of the following criteria: Mini-Mental State Examination (MMSE) ≤ 26, Global Deterioration Scale (GDS) score ≥ 3, or formal documentation of cognitive deficits on neuropsychological testing as determined by clinical consensus. Of the initial 156 patients, 27 were excluded for the following reasons: insufficient clinical follow-up data (*n* = 19), imaging artifacts or incomplete acquisition (*n* = 7), and software processing failure (*n* = 1). The final study cohort comprised 129 patients. There were 95 males and 34 females with an average age of 76.02 ± 5.84 years in this study. The severity of symptoms in all patients was evaluated using the Mini-Mental State Examination (MMSE) and the Global Deterioration Scale (GDS) [23,24]. The clinical diagnosis of AD was established by two experienced neurologists based on the National Institute on Aging and Alzheimer’s Association (NIA-AA) [25]. The clinical data used in this study have not been previously published in any peer-reviewed form and were adapted from preliminary results, which were presented as a poster at the 2025 Society of Nuclear Medicine and Molecular Imaging Annual Meeting [26].

### 2.2. Image Acquisition and Reconstruction

All patients received an intravenous injection of approximately 300 MBq of ^18^F-florbetaben. After a 90 min uptake period, PET/CT imaging was performed using a Discovery MI DR PET/CT scanner (GE Healthcare, Milwaukee, WI, USA). A low-dose CT scan (140 kVp, 200 mAs) was acquired for attenuation correction and anatomical localization, with slice thickness matched to the PET slices (2.79 mm). PET images were acquired in 3D mode over 15 min. Reconstruction was performed using a FORE-iterative algorithm with 20 subsets, 2 iterations, and a post-reconstruction Gaussian filter of 2.14 mm full width at half maximum (FWHM).

### 2.3. Image Processing

All PET/CT images were reviewed for quality control before quantitative analysis. ^18^F-florbetaben PET/CT images obtained from each patient were loaded, and the image quality was visually confirmed. Images passing visual quality assessment were analyzed using three commercially available software platforms: MIMneuro (version 6.9.8, MIM Software Inc., Cleveland, OH, USA), CortexID Suite (version 2.1, GE Healthcare, Marlborough, MA, USA), and Neurophet SCALE PET (version 1.6, Neurophet Inc., Seoul, Republic of Korea). For each patient, one PET/CT dataset was processed by all three tools to generate semi-quantitative SUVRs for amyloid burden. The three software tools available at the hospital were compared with each other, and their features are listed in Table 1. It also included regulatory clearance information accompanied by brief descriptions of their significance for clinical applicability and regional authorization. The brain regions of SUVR that each software could measure were different, and among them, the SUVR values of six specific brain regions (anterior cingulate gyrus, inferior medial frontal gyrus, lateral temporal lobe, posterior cingulate gyrus, precuneus, and superior parietal lobule) that could be measured by all programs and were known as the main sites of amyloid deposition were recorded. In all analyses, the cerebellum was used as the reference region for SUVR calculation. Two neurologists made the final clinical diagnosis.

### 2.4. Statistical Analysis

All 129 amyloid PET/CT images were processed and quantified using three software tools. Statistical analyses were performed using MedCalc (version 23.2.6, MedCalc Software) and SPSS (version 21.0, IBM Corp., Armonk, NY, USA). Inter-software agreement in SUVRs was assessed using Pearson correlation coefficients and the intraclass correlation coefficient (ICC). ICCs were interpreted using the following thresholds: poor (<0.5), moderate (0.5–0.75), good (0.75–0.9), and excellent (>0.9), as recommended by Koo and Li [27]. Differences in regional SUVRs between software platforms were evaluated using paired or independent *t*-tests as appropriate. Receiver operating characteristic (ROC) curve analysis was conducted to assess the diagnostic performance of each software in identifying AD, using clinical diagnosis as the reference standard. A *p*-value < 0.05 was considered statistically significant.

## 3. Results

Of the 129 patients included in the final analysis, 39 (30.5%) were clinically diagnosed with AD by two board-certified neurologists, based on established diagnostic criteria for AD. The demographic and cognitive characteristics are shown in Table 2. There was no significant difference in gender, age, and clinical cognitive scales between the AD and non-AD groups.

To investigate differences in amyloid burden between patients with AD and those without AD, independent t-tests were conducted to compare the SUVRs across six predefined brain regions. The brain regions included the anterior cingulate gyrus, inferior medial frontal gyrus, lateral temporal lobe, posterior cingulate gyrus, precuneus, and superior parietal lobule, all of which are known to be susceptible to amyloid deposition in AD pathology. For each software tool, the comparison between AD and non-AD groups revealed statistically significant differences in SUVR values across all six regions, with *p*-values less than 0.001 (Table 3). Specifically, SUVRs were consistently and significantly higher in the AD group compared to the non-AD group, reflecting increased amyloid uptake in regions typically associated with AD. These findings demonstrated that despite potential differences in processing algorithms and region-of-interest (ROI) segmentation methods, each software tool was able to effectively differentiate between AD and non-AD patients based on quantitative SUVR measurements. The consistent pattern of increased SUVRs in the AD patients supports the diagnostic utility of regional amyloid quantification, reinforcing the role of quantitative analysis in distinguishing AD from other causes of cognitive impairment.

**Figure 1:**
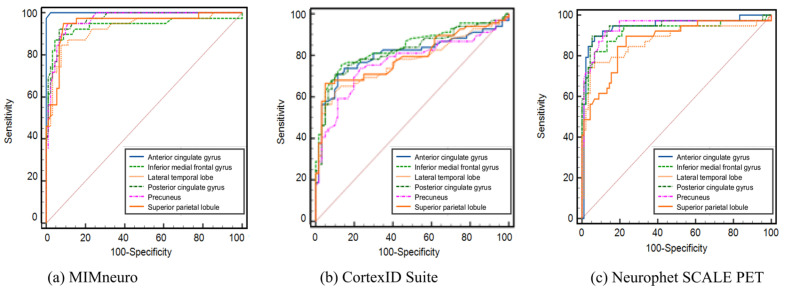
Receiver operating characteristic (ROC) curve analysis for diagnosing Alzheimer’s dementia based on standardized uptake value ratios (SUVR) using (**a**) MIMneuro, (**b**) CortexID Suite, and (**c**) Neurophet SCALE PET software. Area under the curve (AUC) values for each region are listed in Table 4. The highest area under the curve (AUC) was observed in the anterior cingulate gyrus with MIMneuro (AUC = 1.000).

Furthermore, receiver operating characteristic (ROC) curve analyses were performed to assess the discriminative power of each software in identifying AD based on regional SUVRs (Table 4 and Figure 1). All three software tools demonstrated generally high discriminative ability, with area under the curve (AUC) values ranging from 0.767 to 1.000. Among the software tools, MIMneuro showed the highest diagnostic accuracy, achieving an AUC of 1.000 in the anterior cingulate gyrus and 0.969 in the posterior cingulate gyrus, indicating excellent classification performance. Neurophet SCALE PET also showed strong diagnostic performance, with AUC values exceeding 0.90 in most regions. In contrast, the CortexID Suite showed comparatively lower diagnostic accuracy, with AUC values consistently below 0.90.

**Figure 2:**
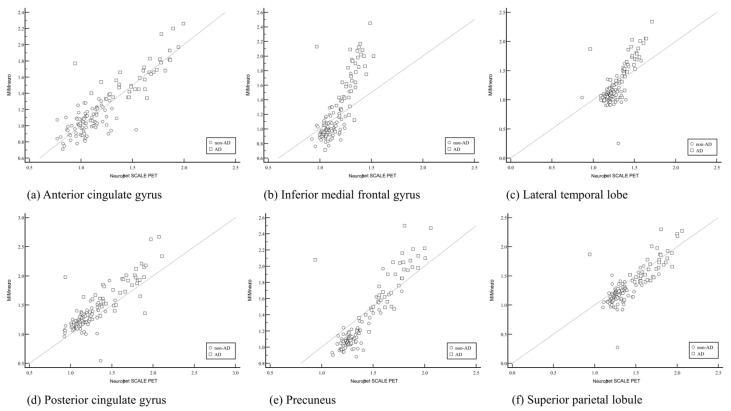
Pearson correlation coefficients of standard uptake value ratio (SUVR) values between MIMneuro and Neurophet SCALE PET for six specific brain regions: (**a**) anterior cingulate gyrus; (**b**) inferior medial frontal gyrus; (**c**) lateral temporal lobe; (**d**) posterior cingulate gyrus; (**e**) precuneus; (**f**) superior parietal lobule. Significant positive correlations were observed for all regions, with correlation coefficients ranging from r = 0.715 to 0.865 (all *p* < 0.001).

**Figure 3:**
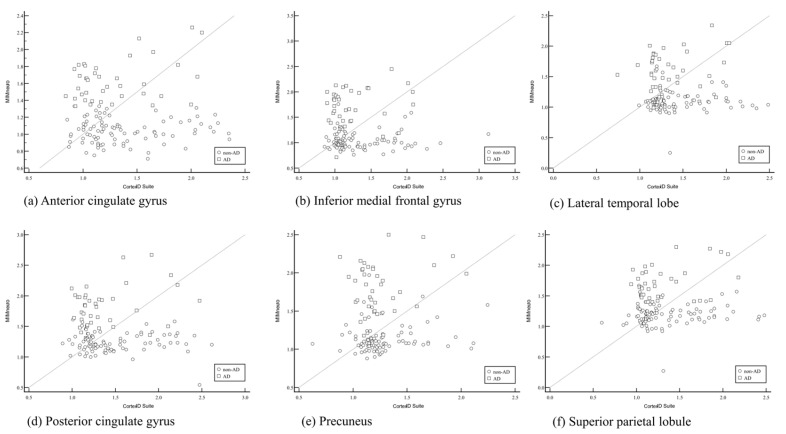
Pearson correlation coefficients of standard uptake value ratio (SUVR) values between MIMneuro and CortexID Suite for six specific brain regions: (**a**) anterior cingulate gyrus; (**b**) inferior medial frontal gyrus; (**c**) lateral temporal lobe; (**d**) posterior cingulate gyrus; (**e**) precuneus; (**f**) superior parietal lobule. No significant correlation was observed in any brain regions.

The SUVRs from six predefined brain regions were compared to evaluate the consistency and agreement between them using Pearson correlation analysis. The results demonstrated a moderate-to-strong positive correlation between SUVRs obtained from MIMneuro and those from Neurophet SCALE PET across all six predefined brain regions. Specifically, the correlation coefficients (r) ranged from 0.715 to 0.865, all of which were statistically significant (*p* < 0.001) (Table 5 and Figure 2). These results indicate a relatively high level of agreement between MIMneuro and Neurophet SCLAE PET, suggesting that these two software tools provide comparable SUV measurements in patients with cognitive impairment. The strength of the correlation implies that both tools detect similar patterns of amyloid uptake in cortical regions relevant to Alzheimer’s pathology. However, the CortexID Suite exhibited poor correlation with both MIMneuro and NEurophet SCALE PET across all analyzed brain regions. The correlation coefficients in these comparisons were low and could not show any statistical significance (*p* > 0.05). Those results indicate a lack of agreement in SUV values derived from CortexID compared to the other two software tools (Figure 3). Overall, these results suggest that while MIMneuro and Neurophet SCALE PET demonstrate high concordance in regional SUVR estimation, the CortexID Suite shows substantial variability and inconsistency when compared with the other tools. Additionally, we evaluated the relationship between regional SUVRs and clinically assessed cognitive function, as measured by the MMSE and the GDS. Among the three software tools, only the CortexID Suite demonstrated statistically significant weak-to-moderate negative correlations between regional SUVRs and MMSE scores (r = −0.359 to −0.271). These findings suggest that increased SUVRs in the CortexID Suite were associated with poorer cognitive performance. However, no significant correlations were observed between MMSE or GDS scores and SUVRs obtained from either MIMneuro or Neurophet SCALE PET.

**Figure 4:**
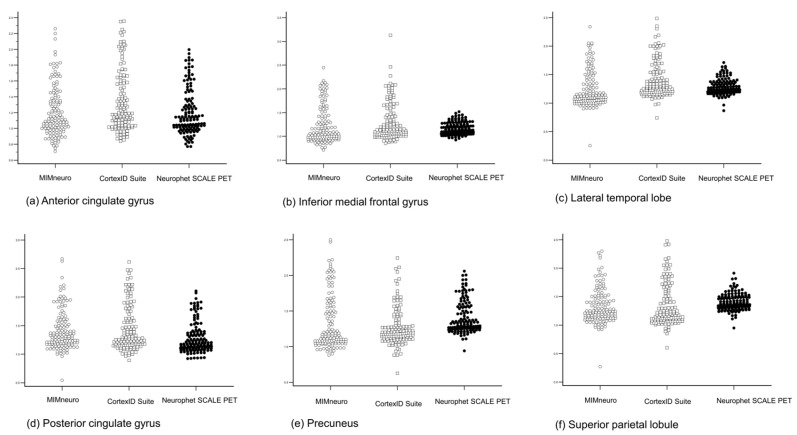
Regional standard uptake value ratio (SUVR) distributions from three amyloid PET quantification software tools—MIMneuro, CortexID Suite, and Neurophet SCALE PET—across six brain regions: (**a**) anterior cingulate gyrus; (**b**) inferior medial frontal gyrus; (**c**) lateral temporal lobe; (**d**) posterior cingulate gyrus; (**e**) precuneus; and (**f**) superior parietal lobule. Each dot represents an individual measurement. The spread of SUVR values illustrates regional variability and differences in quantification values across software tools.

To evaluate the reliability, we performed an ICC analysis. The ICC analysis revealed moderate agreement in several brain regions. The anterior cingulate gyrus demonstrated an ICC of 0.502 (*p* < 0.001), indicating moderate reliability in SUVR measurements across the three tools (Figure 4). Similarly, the posterior cingulate gyrus and precuneus showed ICC values of 0.521 (*p* < 0.001) and 0.565 (*p* < 0.001), respectively. These findings suggest that, despite methodological differences, the software tools provide relatively consistent quantification of amyloid burden in some brain regions.

In contrast, the ICC values were notably lower in other brain regions. The inferior medial frontal gyrus showed an ICC of 0.291, while the lateral temporal lobe and superior parietal lobule exhibited ICC values of 0.334 and 0.418, respectively. These lower ICC values suggest considerable variability in SUVR measurements between the software tools within these regions.

## 4. Discussion

This study compared inter-software differences, correlations, reliability, and diagnostic performance among three commercial quantitative analysis tools for ^18^F-florbetaben PET/CT in patients with cognitive impairment. Many commercial software tools have been applied in the quantitative analysis of amyloid PET. These software tools not only enhance confidence in visual interpretation, reduce inter-reader variability, and present more objective data, but also help clinicians assess the extent of amyloid plaque accumulation and determine whether a patient is a suitable candidate for specific Alzheimer’s treatments. There are more than five commercially available software tools, some of which are primarily used for research purposes. The development of numerous software tools reflects the utility of quantitative analysis in assessing amyloid PET results [11,28,29,30]. Previous research has examined comparisons between commercial software tools for the quantitative evaluation of amyloid PET. Curry et al. (2019) compared Hermes and Syngo.VIA, reporting high sensitivity for Hermes but lower specificity, concluded that interpretation requires experienced readers [31]. More recently, Pemberton et al. (2023) compared several software tools—MIMneuro, NeuroQ, BRASS, and CortexID—on 80 subjects, finding high correlations and reliability in quantifying [^18^F]flutemetamol PET [14]. As can be seen from the various research results, different software tools can yield different results; therefore, it is necessary to compare them with existing tools before using a new software tool. In addition, this comparative analysis is meaningful because no comparative study has included Neurophet SCALE PET to date.

In this study, we conducted a comparative analysis of amyloid PET quantification across MIMneuro, CortexID Suite, and Neurophet SCALE PET, focusing on their ability to differentiate between patients with AD and non-AD patients using SUVRs from six brain regions (anterior cingulate gyrus, inferior medial frontal gyrus, lateral temporal lobe, posterior cingulate gyrus, precuneus, and superior parietal lobule). Independent t-tests revealed statistically significant group differences (*p* < 0.001) across all regions and all software tools, with the AD group showing markedly higher SUVRs compared to the non-AD group. These results are clinically meaningful, as they validate the ability of semi-quantitative software tools to complement visual assessment in the differential diagnosis of cognitive impairment. Although visual amyloid PET evaluation images have shown approximately 90% accuracy in advanced clinical and end-of-life cases [32,33], their reliability decreases in heterogeneous populations, especially in the early or mild cognitive impairment stage, where amyloid deposition may be subtle or focal. In such cases, quantitative software tools offer objective, reproducible metrics to supplement visual reads. Also, the use of quantification software tools can enhance diagnostic confidence, particularly when evaluating atypical presentations or monitoring disease progression. To further explore the diagnostic performance of the software tools in diagnosing AD, the SUVRs were analyzed using ROC curve analysis. It further highlights the diagnostic value of regional SUVRs in differentiating AD from non-AD patients. Among the three software tools evaluated, MIMneuro demonstrated the highest overall diagnostic performance, particularly in the anterior and posterior cingulate gyri, which are regions known to accumulate amyloid early in the disease process. Overall, these findings support the clinical utility of the software tools in diagnosing AD.

However, despite their diagnostic capabilities, inter-software correlation and reliability were not uniformly high. In this study, we compared regional SUVR values derived from three commercially available amyloid PET quantification software tools in patients with cognitive impairment. Our findings highlight significant variability in quantitative outputs between software tools, with important implications for clinical interpretation. A key finding was the moderate-to-strong correlation between SUVRs derived by MIMneuro and Neurophet SCALE PET across six brain regions. The observed Pearson correlation coefficients (r = 0.715–0.865, all *p* < 0.001) indicate a high degree of agreement, suggesting that both tools produce broadly comparable quantitative results when evaluating amyloid burden. In contrast, the CortexID Suite demonstrated poor correlation with both MIMneuro and Neurophet, with no statistically significant association in any of the six regions. In CortexID Suite, unusually wide SUVR ranges observed in the lateral temporal and superior parietal lobule regions were partially driven by outlier cases, as well as differences in regional parcellation and intensity normalization. These factors may reflect underlying variability in the algorithm’s segmentation and scaling approaches. Further investigation is needed to determine the reproducibility of these values across populations and platforms. The relatively lower diagnostic performance and weaker correlation of the CortexID Suite with other platforms may stem from several factors. First, differences in anatomical templates and segmentation algorithms may result in regionally inconsistent SUVR values compared to other software. Second, the normative database used by CortexID Suite may differ in terms of population demographics, acquisition protocols, or image preprocessing pipelines. Lastly, fixed region definitions and limited flexibility in reference region selection within the CortexID Suite may contribute to variability in diagnostic performance. These methodological distinctions should be taken into account when interpreting results across software tools. Interestingly, SUVR values from CortexID Suite showed a weak-to-moderate negative correlation with MMSE scores, suggesting some clinical utility in tracking cognitive decline. However, this was not consistently observed across the other software tools. The correlation between SUVRs from CortexID Suite and MMSE scores, not observed with other software tools, may be attributed to several factors. These include differences in segmentation algorithms, such as region boundary definition; variations in reference region implementation; and the use of distinct normative databases. Additionally, discrepancies in image preprocessing pipelines and susceptibility to measurement noise could also influence the strength of correlation with MMSE. Further comparative studies are warranted to elucidate the impact of these methodological differences on clinical interpretation.

Additionally, the ICC results highlight that while certain brain regions demonstrate moderate inter-software reliability, others show limited concordance, underscoring the need for cautious interpretation of SUVR values, particularly when comparing results across different software tools. This discrepancy may be attributable to methodological differences in imaging processing pipelines, region-of-interest (ROI) definitions, or reference region normalization strategies unique to the CortexID Suite. These findings underscore the importance of recognizing inter-software variability in quantitative amyloid PET analysis. While visual interpretation remains the standard in many clinical settings, semi-quantitative tools are increasingly used to support diagnostic confidence, assess disease progression, and evaluate treatment response. However, inconsistencies across software tools may introduce misclassification, particularly in borderline cases or when comparing data across institutions or longitudinal time points using different tools. Caution is warranted when using or interpreting SUVRs in comparative analyses, as their quantitative results may not align with those from other software tools.

This study has several limitations. First, the normal databases used by each software tool differ substantially in terms of their control populations, imaging acquisition protocols, and definitions of reference regions. These variations may contribute to inter-software discrepancies in SUVR calculations, thereby limiting direct comparability between software tools. Second, our comparative analysis focused exclusively on SUVR values, without incorporating other quantitative parameters such as Z-scores or Centiloid scaling. Although all three platforms support Centiloid scaling or Z-scores, these values were not consistently available across all cases due to differences in software configuration and institutional setup. Since these standardized approaches are increasingly recognized for improving inter-platform comparability and facilitating clinical interpretation of amyloid PET. Centiloid scaling, in particular, enables direct comparison across tracers and imaging centers by mapping SUVR values to a universal scale. Z-scores, which express regional tracer uptake relative to a normative database, also support individualized interpretation. However, differences in anatomical segmentation, tracer calibration procedures, and database characteristics currently limit uniform adoption across commercial tools. Further studies should prioritize direct Centiloid or Z-scores comparison to facilitate cross-platform harmonization and to support broader clinical implementation. Third, SUVR normalization was uniformly performed using the cerebellum as the reference region. It is well known that the choice of reference region significantly influences SUVR values and may affect reproducibility and consistency. Although cerebellar normalization was consistently applied in this study, we acknowledge that alternative reference regions, such as the pons or subcortical white matter, may yield different SUVR values. Further studies should evaluate alternative reference regions to better understand their impact on quantitative outcomes. Fourth, all imaging data were acquired from a single PET/CT scanner at a single institution, and reproducibility across different scanners was not evaluated. While this uniformity allowed for controlled comparison of software performance, it may limit the external validity of our findings. Further studies involving multicenter data and multiple scanner types are warranted to assess the generalizability. Lastly, one limitation of this study is the male predominance in the cohort, which reflects the demographics of the veteran population from which participants were recruited. This gender imbalance may affect the generalizability of our findings to more diverse clinical settings, and future studies should aim to include more balanced populations.

## 5. Conclusions

This study demonstrates that MIMneuro, CortexID Suite, and Neurophet SCALE PET provide clinically applicable semi-quantitative tools for distinguishing AD from non-AD. While their overall diagnostic performances are comparable, we observed notable inter-software variability in SUVR quantification. These differences likely originate from variations in reference region definitions, segmentation algorithms, and normative databases used across software tools. Furthermore, the observed differences in regional SUVR values and the modest inter-software agreement suggest that results should not be used interchangeably across software tools. Careful interpretation is warranted, particularly when the value is near diagnostic thresholds. To minimize diagnostic inconsistencies, consistent use of a single analysis software tool or broader standardization efforts is strongly recommended.

## Figures and Tables

**Table 1:** Comparison of Amyloid PET analysis tools for ^18^F-florbetaben.

Feature	MIMneuro	CortexID Suite	Neurophet SCALE PET
Developer	MIM Software Inc.	GE Healthcare	Neurophet Corp.
Software Version	v6.9.8	v2.1	v1.6
Regulatory Clearance			
FDA Clearance	Yes	Yes	Yes
CE Marking	Yes	Yes	Yes
MFDS Approval	Yes	Yes	Yes
Tracer	^18^F-Florbetaben, ^18^F-Florbetapir, others	^18^F-Florbetaben, ^18^F-Florbetapir, others	^18^F-Florbetaben, ^18^F-Florbetapir, others
Centiloid scaling	Yes	Yes	Yes
Quantification	SUVR, Z-score	SUVR, Z-score	SUVR, Z-score
Normative Database	Age-matched, scanner-independent	Age-matched, scanner-dependent	Age-matched, multi-ethnic normative database
Normative Database Size	~74 cognitively normal adults	~50–70 cognitively normal controls	~100 cognitively normal adults
Automated anatomical segmentation	Yes	Yes	Yes
Anatomical Template	Talairach-like (proprietary)	MNI152 (Montreal Neurological Institute)	Neurophet proprietary MRI template
Segmentation Method	Atlas-based segmentation	Atlas-based segmentation	Atlas-based segmentation
Visual interpretation support	Yes	Yes	Yes
Quality Control Tools	Visual verification	Visual verification	Visual review and structured QC reports

SUVR = Standardized uptake value ratio; FDA clearance indicates that the software has been reviewed and authorized for clinical use by the U.S. Food and Drug Administration, typically under the 510(k) pathway for medical devices. CE marking signifies conformity with health, safety, and environmental protection standards in the European Economic Area, allowing marketing and clinical use in EU countries. MFDS approval denotes authorization for clinical application in South Korea by the Ministry of Food and Drug Safety. These regulatory designations support clinical implementation but may vary in terms of the depth of validation and required evidence for approval in each region.

**Table 2:** Comparison of demographic and cognitive characteristics between Alzheimer’s dementia (AD) and non-AD Groups.

	AD (*n* = 39)	Non-AD (*n* = 90)	*p*-Value
Gender (male vs. female)	27 vs. 12	68 vs. 22	0.585
Age	75.94 ± 5.86	76 ± 5.86	0.958
MMSE	24.07 ± 4.50	24.06 ± 4.49	0.991
GDS	3.97 ± 2.91	3.93 ± 2.78	0.941

AD = Alzheimer’s dementia; MMSE = Mini-Mental State Examination; GDS = Global Deterioration Scale.

**Table 3:** Regional SUVR values (mean ± standard deviation) in Alzheimer’s dementia (AD) and non-AD groups across three quantitative software programs.

Brain Region	Software	AD (*n* = 39)	Non-AD (*n* = 90)	*p*-Value
Anterior cingulate gyrus	MIMneuro	1.37 ± 0.34	1.19 ± 0.42	<0.001
	CortexID Suite	1.54 ± 0.42	1.13 ± 0.19	<0.001
	Neurophet SCALE PET	1.34 ± 0.31	1.16 ± 0.27	<0.001
Inferior medial frontal gyrus	MIMneuro	1.35 ± 0.45	1.18 ± 0.35	<0.001
	CortexID Suite	1.50 ± 0.44	1.08 ± 0.15	<0.001
	Neurophet SCALE PET	1.20 ± 0.15	1.14 ± 0.11	<0.001
Lateral temporal lobe	MIMneuro	1.34 ± 0.33	1.22 ± 0.29	<0.001
	CortexID Suite	1.55 ± 1.24	1.24 ± 1.34	<0.001
	Neurophet SCALE PET	1.31 ± 0.15	1.27 ± 0.13	<0.001
Posterior cingulate gyrus	MIMneuro	1.54 ± 0.37	1.37 ± 0.35	<0.001
	CortexID Suite	1.65 ± 0.43	1.21 ± 0.18	<0.001
	Neurophet SCALE PET	1.40 ± 0.31	1.25 ± 0.25	<0.001
Precuneus	MIMneuro	1.47 ± 0.41	1.29 ± 0.37	<0.001
	CortexID Suite	1.34 ± 0.31	1.19 ± 0.18	<0.001
	Neurophet SCALE PET	1.49 ± 0.26	1.39 ± 0.21	<0.001
Superior parietal lobule	MIMneuro	1.43 ± 0.33	1.30 ± 0.28	<0.001
	CortexID Suite	1.51 ± 1.14	1.14 ± 0.15	<0.001
	Neurophet SCALE PET	1.44 ± 0.17	1.39 ± 0.14	<0.001

SUVR = Standardized uptake value ratio; AD = Alzheimer’s dementia.

**Table 4:** Comparison of diagnositc performance across brain regions using MIMneuro, CortexID Suite, and Neurophet SCALE PET.

Brain Region	Software	Cut-off Value	Sensitivity	Specificity	AUC
Anterior cingulate gyrus	MIMneuro	≤1.31	97.78	100.00	1.000
	CortexID Suite	≤1.245	72.41	85.71	0.822
	Neurophet SCALE PET	≤1.267	93.33	89.74	0.945
Inferior medial frontal gyrus	MIMneuro	≤1.3	95.56	87.18	0.937
	CortexID Suite	≤1.135	70.11	88.10	0.831
	Neurophet SCALE PET	≤1.216	93.33	82.05	0.923
Lateral temporal lobe	MIMneuro	≤1.28	92.22	84.62	0.927
	CortexID Suite	≤1.335	74.71	73.81	0.765
	Neurophet SCALE PET	≤1.344	93.33	76.92	0.876
Posterior cingulate gyrus	MIMneuro	≤1.46	93.33	92.31	0.969
	CortexID Suite	≤1.28	70.11	85.71	0.813
	Neurophet SCALE PET	≤1.344	93.33	76.92	0.876
Precuneus	MIMneuro	≤1.32	88.89	94.87	0.966
	CortexID Suite	≤1.245	78.16	69.05	0.768
	Neurophet SCALE PET	≤1.407	87.78	92.31	0.944
Superior parietal lobule	MIMneuro	≤1.37	91.11	94.87	0.948
	CortexID Suite	≤1.29	79.31	73.81	0.804
	Neurophet SCALE PET	≤1.393	76.67	89.74	0.876

AUC = Area under the curve.

**Table 5:** Pearson correlation coefficients (r) for regional standard uptake value ratio (SUVR) comparisons between software tools.

Brain Region	MIMneuro vs. CortexID Suite	MIMneuro vs. Neurophet SCALE PET	CortexID Suite vs. Neurophet SCALE PET
Anterior cingulate gyrus	0.004	0.865 *	0.030
Inferior medial frontal gyrus	−0.025	0.819 *	0.032
Lateral temporal lobe	−0.016	0.758 *	0.013
Posterior cingulate gyrus	−0.041	0.729 *	0.088
Precuneus	0.051	0.828 *	0.068
Superior parietal lobule	0.080	0.715 *	0.120

* Statistically significant correlation (*p* < 0.001).

## Data Availability

The data that support the findings of this study are available from the corresponding author M.C., upon reasonable request.
